# Inquiring Pant‐Hoots in Wild Chimpanzees and the Role of Social Bonds and Group Cohesion

**DOI:** 10.1002/ajp.70092

**Published:** 2025-12-12

**Authors:** Lara Michelle Southern, Tobias Deschner, Simone Pika

**Affiliations:** ^1^ Comparative BioCognition, Institute of Cognitive Science Osnabrück University Osnabrück Lower Saxony Germany; ^2^ Max Planck Institute for Evolutionary Anthropology Leipzig Saxony Germany

**Keywords:** communication, long‐distance calls, *Pan troglodytes troglodytes*, turn‐taking, vocalizations

## Abstract

The evolution of human language remains a puzzle, with comparative approaches focusing on vocalizations, gestures, bimodal combinations, and, most recently, social interaction and turn‐taking. The latter is characterized by cooperative, reciprocal exchanges of alternating short bursts of information among interactants. Some hallmarks of human conversational turn‐taking have been found in other primate species, suggesting a possible specialization of great apes in gestural rather than vocal turn‐taking. However, relatively little is known about the vocal turn‐taking abilities of great apes. Here, we conducted a systematic, quantitative study on vocal exchanges of adult male chimpanzees (*Pan troglodytes*) living in a habituated community in the Loango National Park, Gabon. We focused on pant‐hoots, the typical long‐distance calls of chimpanzees, which have been argued to function in some contexts as question‐and‐answer‐like exchanges, referred to as “inquiring pant‐hoots” (IPHs), a term coined by Goodall (1986). We collected a comprehensive data set over a period of 16 months (January–May 2019; November 2019–November 2020) resulting in a total of 1747 pant‐hoots of ten adult males. We analyzed the data with a special focus on general pant‐hoot patterns, criteria for IPHs, social factors, and temporal organization. Overall, general calling frequency was highest in males with high social ranks, in larger parties, and during periods of increased fission and fusion. Twenty percent of calls qualified as IPHs and were positively correlated with smaller party size, higher fission–fusion rates, and the absence of close social partners. Temporal patterns were influenced by social bond strength, the presence of drumming, and an avoidance of overlap. Our findings add to the growing evidence of complex vocal turn‐taking abilities in nonhuman primates, contradicting the notion of a specialization in gestural rather than vocal turn‐taking for chimpanzees and possibly other great apes. They also emphasize the role of long‐distance vocalizations for species in fission–fusion societies and visually dense environments.

## Summary

- Chimpanzees produce “Inquiring Pant‐Hoots” (IPHs) more frequently in social contexts associated with small parties and high fission–fusion rates, consistent with a role in group coordination.
- IPHs are characterized by distinct behaviors indicating active listening and waiting for responses, akin to vocal turn‐taking interactions observed in other primate species.
- The dynamics behind this long‐distance communication system coordinating traveling parties and individuals are affected by a range of social factors such as rank, social bonds, party composition, and fission–fusion rates.

## Graphical abstract

Traveling chimpanzees engage in structured vocal turn‐taking. Inquiring pant‐hoots are followed by responses within two distinct reply‐time windows, and males with stronger social bonds respond in the faster window more frequently than weakly bonded partners.

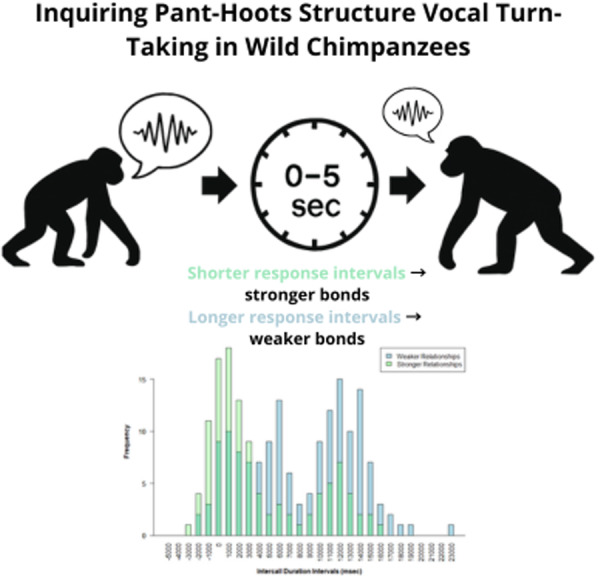

## 1 Introduction

Understanding the evolution of language requires exploring the similarities and differences between human and nonhuman communication systems (Arbib et al. 2008; Liebal et al. 2013; Partan and Marler 1999; Seyfarth and Cheney 2003). Comparative studies offer key insights into this process, helping to uncover the evolutionary roots of language (Fitch 2010). Most research focused on bird song (Aamodt et al. 2020) and vocalizations of nonhuman primates (hereafter, primates), specifically loud calls (White et al. 2015). Overall, previous studies showed that loud calls are predominantly used in long‐distance communication, and are the most distinctive vocalizations across species (Mitani and Stuht 1998; Kitchen et al. 2003). They serve several different functions, including mitigating predation risk (Cäsar and Zuberbühler 2012), competing with other individuals or groups (Wich and Nunn 2002), as well as cooperative ones such as maintaining group cohesion (Teixeira da Cunha and Byrne 2009) and forming and nurturing social bonds (Searcy and Andersson 1986).

Two key hypotheses about vocal communication complexity have recently gained research attention. The first, the “social complexity hypothesis,” suggests that social complexity drives vocal complexity, especially in primates (Freeberg et al. 2012; Seyfarth et al. 2005). However, researchers are challenged with defining and measuring “complexity” for both social and communicative aspects. Furthermore, limitations also stem from a research bias toward emitter‐centered data collection, while communicating and socializing are inherently acts of interaction (Levinson and Enfield 2006). The second hypothesis, the “Interaction‐engine hypothesis,” suggests that human complex communication arose from a unique capacity for social interaction. This entails characteristics like face‐to‐face engagement, mutual gaze, and cooperative turn‐taking. The conversational turn‐taking system underlying language involves reciprocal, alternating bursts of information and adheres to universal properties (Sacks et al. 1974), with minimal overlap and stable turn gaps across languages (Stivers et al. 2009). This infrastructure has recently gained considerable research attention by comparative researchers, as some primates seem to exhibit features akin to human conversational turn‐taking (Abreu and Pika 2022; Takahashi et al. 2013; Fröhlich et al. 2016; Pika et al. 2018). Levinson (2016) also suggested that great apes may have specialized in gestural rather than vocal turn‐taking, offering a perspective that reconciles the “gesture‐first hypothesis” with the co‐evolution of gesture and speech (Levinson and Holler 2014).

Recent studies have highlighted parallels between human social actions during conversations and nonhuman great ape gestural turn‐taking (*Pan troglodytes* and *Pan paniscus*: Fröhlich et al. 2016; Kolff and Pika 2025; Rossano 2013; *Pongo abelii*, Rossano and Liebal 2014). However, research on great apes' vocal turn‐taking remains somewhat inconsistent (Cornec et al. 2022; Levréro et al. 2019; Schamberg et al. 2016; Arcadi 2000; Luef et al. 2016; Lemasson et al. 2018; Pougnault et al. 2020). For instance, Schamberg and colleagues (2016) showed that bonobos at the LuiKotale field site, DRC, used call‐and‐answer exchanges to coordinate reunions and may be able to modify the acoustic structure of call combinations to mark response calls. In contrast, Arcadi (2000) found that adult chimpanzee males of the Kanyawara community, Kibale National Park, Uganda did not respond to the majority of calls heard. He concluded that chimpanzees' vocal interactions lack rudimentary features of human conversational turn‐taking. Discrepancies across studies may result from methodological differences, such as definitions, observation periods, and vocalization type selection, impeding comparative analysis (Pika et al. 2018).

**Figure 1:**
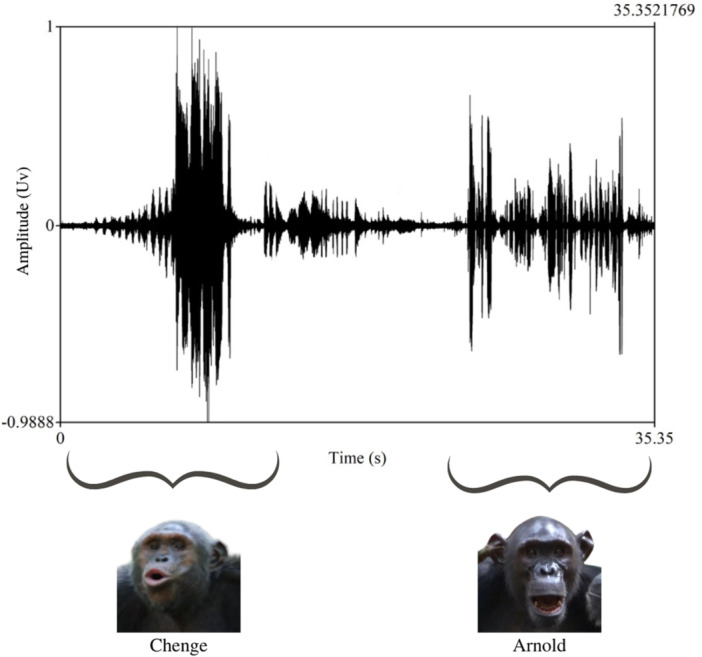
The figure shows a spectrogram of an exemplary IPH and subsequent response between two adult male chimpanzees, Chenge and Arnold, as a function of amplitude and time (in s).

To gain a more comprehensive understanding of vocal turn‐taking skills of great apes and to add to the current debate, we studied vocal interactions of central chimpanzees (*Pan troglodytes troglodytes*) living in a habituated community in the Loango National Park in Gabon. We specifically focused on their species‐typical long‐distance vocalization—the pant‐hoot (Goodall 1986)—which can be heard over distances of up to one km depending on habitat (Reynolds and Reynolds 1965). Pant‐hoot production is often accompanied by drumming, which involves pounding one or both hands and/or feet against various substrates, including tree buttresses, resulting in low‐frequency sounds that travel further than pant‐hoots alone (Babiszewska et al. 2015). Several functions for pant‐hoots have been proposed, ranging from attracting others to food sources (Reynolds and Reynolds 1965), conveying the caller's identity and social status (Clark and Wrangham 1994; Mitani and Nishida 1993), territory defense (Mitani and Rodman 1979), and maintaining cohesion among dispersed group members (Boesch 1991; Eckhardt et al. 2015). Studies have also shown significant variation in the acoustic structure of pant‐hoots both within (Brima et al. 2023; Desai et al. 2022) and between populations (Crockford et al. 2004; Mitani et al. 1992). Whether group “dialects” exist remains, however, an open question (Desai et al. 2022; Mitani et al. 1999). Additionally, demographic and social factors have been found to influence pant‐hoot dynamics (Fedurek et al. 2014; Mitani and Nishida 1993; Uhlenbroek 1995). More relevant for the present study, Goodall (1986) noted that Eastern chimpanzees (*Pan troglodytes schweinfurthii*) at Gombe, Tanzania, use different pant‐hoot types (see also: Clark and Wrangham 1993; Reynolds and Reynolds 1965), including the “Inquiring Pant‐Hoot” (IPH). She suggested that this call type may initiate call‐and‐answer exchanges between two or more out‐of‐sight individuals (Figure 1) and involves six shared characteristics (see Section 2) (Goodall 1986).

The present study revisited Goodall's (1986) hypothesis that chimpanzees use pant‐hoots when traveling to coordinate movement, fusion, and inquire about nearby group members. To do so, we analyzed pant‐hoot patterns of ten adult males, with a special focus on call context, caller identity, party size and composition, and fission–fusion dynamics. Furthermore, we examined whether travel‐associated pant‐hoots met Goodall's criteria and how factors such as age, rank, social bonds, and drumming presence influenced call production and intervals. Lastly, we explored the time windows between repeated pant‐hoots of a caller receiving no response to understand which durations chimpanzees perceive as indicating “no one around” versus “no one answering.”

## 2 Methods

### 2.1 Ethics Statement

The study was purely observational and noninvasive and adhered to national and institutional animal care guidelines. In accordance with the German Animal Welfare Act (1998), the study was classified as nonanimal research and did not require ethical approval. Observers followed strict hygiene protocols, including a five day quarantine, mandatory face masks, and a minimum distance of eight ms to the chimpanzees to reduce disease transmission and disturbance. Our research complied with the legal requirements of Gabon, following the recommendations of the “Animals (Scientific Procedures) Act 1986” and the principles of “Ethical Treatment of Non‐Human Primates,” as stated by the American Society of Primatologists, the Agence Nationale des Parcs Nationaux (ANPN), and the Centre National de la Recherche Scientifique et Technique of Gabon (CENAREST). Permission and permits to conduct research in the Loango National Park were obtained from the ANPN and the CENAREST in Gabon.

### 2.2 Study Site and Subjects

Behavioral data were collected of central chimpanzees living in the Rekambo community, Loango National Park, Gabon (1°51’ S and 9°15’ E). This National Park comprises a mosaic of different habitat types, including coastal forests and savannahs, multiple lowland swamps fed by a lagoon, and heterogeneous tropical rainforest throughout. The community consisted of about 45 individuals, including infants, juveniles, subadult, and adult individuals, at the start of the data collection period in January 2019. We specifically focused on the behavior and pant‐hoot production of adult males because of their habituation levels to human researchers and their greater gregariousness and pant‐hoot output. These are leading to more frequent involvement in fission–fusion decisions compared with other sex and age classes (Mitani 2009). Following the classification of Goodall (1986), males were defined as “adults” when their estimated age was ≥ 15 years old. The mean age across the subjects was 21.8 (± SD = 3.45) years. Age, rank, observation hours, and call counts are listed in the Supporting Information S1: Table S1.

### 2.3 Data Collection

L. M. S. collected a comprehensive behavioral data set on ten adult males over a nonconsecutive time period of 16 months (January–May 2019 and November 2019–November 2020). During an initial three‐month pilot period, L. M. S. trained to reliably distinguish between the individual pant‐hoots of the males (see Supporting Information S1: Identity recognition). Focal individuals were followed daily from early morning to evening, resulting in a total of 301 observation days and a mean of 210.1 observation hours (± 74.5) per focal subject. Applying instantaneous scan sampling at 10‐min intervals (Altmann 1974), she recorded the proximity between the focal subject and other individuals (1 m, 5 m, 10 m – total *N* = 3476 scans) and updated the identities of party members—defined here as all individuals within visual range of the focal subject. Behavioral data were entered on a smartphone using Cybertracker (Version 3.51) based on a standardized ethogram for chimpanzee behavior. Pant‐hoot recordings were collected opportunistically within approximately 30 m of the initial caller using a Sennheiser directional microphone (ME66/K6) and an audio recorder (Zoom H4n; 44.1 kHz, 16‐bit). Caller identity was visually confirmed in the field and verified through a structured training and reliability testing procedure (Cohen's *κ* = 0.95; see Supporting Information S1: Methods).

### 2.4 IPH Criteria

We used and adapted the criteria outlined by Goodall (1986) for identifying IPHs as follows: (1) Travel Context: The pant‐hoot occurred during travel (excluding movement within a feeding site or during patrols/intergroup encounters). (2) Waiting and Scanning: The call was accompanied by one or several brief pauses during travel to scan the environment or listen in a particular direction. To ensure that these behaviors reflected deliberate actions rather than incidental glances, calls met this criterion only if at least one instance of waiting, scanning, or listening lasted at least 3 s. (3) Fusion: The caller and responder were in the same party composition within 30 min of the call, defined as having moved into the same party. (4) Drumming: The pant‐hoot was accompanied by buttress drumming (yes/no). To classify a pant‐hoot as an IPH, it had to meet criteria 1 and 2 (travel context and waiting/scanning behavior). The additional features—including fusion within 30 min, drumming, and receiving a vocal response—were recorded for each event but were not required for classification as an IPH. Two additional behaviors noted by Goodall—calling from high ridges and a pitch rise at the call's end—were not collected, respectively analyzed. The former could not be investigated due to the flat terrain of the Rekambo territory, and the latter will be addressed in future acoustic analyses. After each pant‐hoot recording, we also noted any responses and estimated the distance between the caller and the responder (Supporting Information S1: Identity recognition and Speed of sound adjustment).

### 2.5 Age, Rank, Social Bond Strength, Fission–Fusion Rates

We estimated the age of the males using camera trap data from 2006 to 2020 and prior estimates (Estienne et al. 2019). Birthdates were established as the first sighting date minus the initial age estimate. For individuals born after 2000, age estimates are generally accurate to within six months to one year, while individuals born prior may have broader estimates ranging from one to four years. We calculated dominance ranks using interaction matrices based on pant grunts (Goodall 1986) and observed aggression outcomes (Altmann 1974). These matrices were analyzed in MatMan, where we assessed the linearity of the hierarchy using the h′ index (de Vries et al. 1993). Rank positions (scored 1–10) were determined separately for the two data collection periods. Additionally, we calculated the steepness of the hierarchy using the *steepness* function in R (de Vries et al. 2006), with values ranging from 0 (egalitarian) to 1 (despotic). We measured the strength of social bonds using the Dyadic Composite Sociality Index (DSI) across all dyads (Silk et al. 2003), based on rates of grooming and spatial proximity. The DSI score was determined through the following equation, where *G*(A + B) and *P*(A + B) represent the rates of grooming and proximity between partners *A* and *B*, and *G*(A), *G*(B), *P*(A), and *P*(B) denote each individual's mean rate with all other partners (see Supporting Information S1: Dyadic Composite Sociality Index):

$$\text{DSI}\;=\left\{\frac{\left(G_{A+B}/{(G}_{A+B}+G_{A}+G_{B})+(P_{A+B}/{(P}_{A+B}+P_{A}+P_{B}\right)}{2}\right\}$$

We calculated fission–fusion rates by analyzing ten‐min scan samples across each focal observation. For each focal day, we recorded the number of entry or exit events—defined as individuals joining or leaving the focal's party—and divided this by the total number of observation hours to yield a standardized rate (events per hour).

### 2.6 Statistical Analyses

We used generalized linear mixed models (GLMMs) to examine pant‐hoot patterns, the impact of IPHs, and the influence of demographic and social factors. Analyses were conducted in R (version 3.9.1; R Core Team 2022) with the glmer function from the lme4 package (Bates et al. 2015). Model significance was evaluated by comparing each model with a null model lacking test predictors using Likelihood Ratio Tests (LRT) via the ANOVA function (Dobson and Barnett 2018). We assessed multicollinearity using the Variance Inflation Factor (VIF) from the car package, confirming that all values were below 2.3, thus indicating no collinearity issues (Fox and Weisberg 2011).

### 2.7 Model 1a

For this model, we included all pant‐hoots produced in the traveling context (regardless of subtype) to assess how social and contextual factors broadly influence call production during travel. We used a GLMM with a Poisson error distribution and a log link function to evaluate pant‐hoot call rates per focal day. We modeled call counts per focal and included a log‐transformed offset for total observation time to account for variation in observational effort. We specified age, social rank, party size, fission–fusion rate, and the presence of a preferred association partner as fixed effects. The presence of a preferred partner was coded as a binary variable (“Yes”/“No”), with “Yes” assigned when the partner was present during more than 50% of the 10‐min scan samples within a focal day. To test for differential effects across call types, we included interaction terms between pant‐hoot type (inquiring vs. general) and each of the following: party size, fission–fusion rate, and preferred partner presence. To account for repeated observations, we added a random intercept for individual identity.

### 2.8 Model 1b

We used a GLMM with a binomial error structure and a logit link function to examine the likelihood of receiving a vocal response following an IPH. We coded the binary response variable as 1 if the call was followed by a response and 0 if not, based on a sample of 352 calls (260 responses). We specified social rank, caller age, and social bond strength between the caller and potential responder as fixed effects. To account for repeated measures, we added random intercepts for caller identity and, where applicable, recipient identity.

### 2.9 Model 2

We used a GLMM with a Gaussian error structure to analyze intercall intervals and assess the influence of social and acoustic factors on response timings. We measured the onset and offset of all pant‐hoots and their responses. Intercall intervals were calculated as the time from the offset of the initial call to the onset of the response; negative values indicate cases where the response began before the initial call had ended. Fixed effects included social bond strength, drumming presence, caller age, and responder age. To account for repeated observations within pairs, we included a random intercept for dyadic ID, defined as a directional combination of caller and responder (e.g., AB ≠ BA). This approach allowed us to control for individual‐ and pair‐level variation in response timing without overfitting.

To further examine timing differences in vocal exchanges, we conducted a Kolmogorov–Smirnov (K‐S) test to compare the distribution of intercall times—measuring the delay between a call and its response—to the distribution of wait times, which reflect how long a chimpanzee waits before producing a second IPH (Eckhardt et al. 2015).

Effect sizes for key predictors and their 95% confidence intervals were reported to provide context on the magnitude and precision of observed effects.

## 3 Results

### 3.1 Dominance Rank and Party Size

The dominance hierarchy among the adult males of the Rekambo community was highly linear and remained stable across both data collection periods. We tested the linearity of the male rank order using the *h*′ index in MatMan 1.1 (de Vries et al. 1993), which yielded *χ*²₉ = 447.89, *p* < 0.0001, *h*′ = 0.97, *K* = 0.97. Rank order did not vary between study periods one and two. We assessed the steepness of the hierarchy by calculating the slope of the best‐fit line of normalized David's scores (NormDs), resulting in a steepness of 0.93 in period one and 0.90 in period two (de Vries et al. 2006). The average male party size per focal day was 4.2 individuals (± 3.1 SD).

### 3.2 General Pant‐Hoot Rates

**Figure 2:**
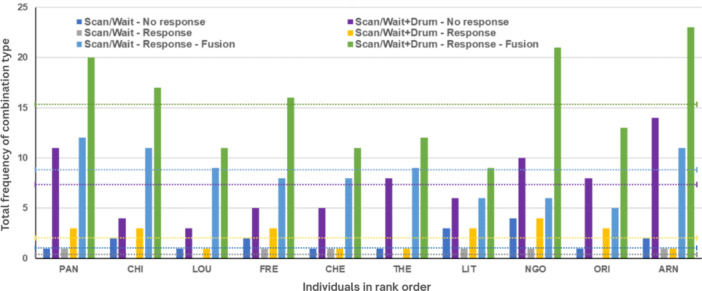
The figure depicts total frequencies of IPH calls made in the traveling context as a function of all tested IPH criteria and caller ID. Dotted lines show the average number of calls observed across each category (see legend). Focal males are shown via their three‐digit abbreviations and in relation to their rank (from highest to lowest from left to right) on the *x*‐axis.

We recorded a total of 1747 pant‐hoot calls. On average, males produced 1.21 pant‐hoots per hour, and 36% of these (*N* = 629) included drumming. Pant‐hoots were produced across four different contexts, most frequently during travel (45%; *N* = 789) and feeding (29%; *N* = 509), followed by resting (18%; N = 315) and during displays (7.5%; *N* = 132). Based on our criteria, a total of 352 calls qualified as IPH calls because they were accompanied by waiting and/or scanning the area (*N* = 352; 100%), drumming (*N* = 250; 71%), receiving a vocal response (*N* = 267; 75%), and resulted in a fusion event (*N* = 238; 67%). The investigated criteria differed in their combinations, with the combination involving all criteria (Drum–Wait/scan–Response–Fusion) being the most frequent (*N* = 153, mean: 15/male), while the reduced combination (Scan/Wait–Response) was rarely observed (*N* = 6; mean: 0.6/males; see Figure 2). Most IPH calls were accompanied by drumming (71%; *N* = 250) while only 24% involved drumming when produced as aresponse call (*N* = 64). After an IPH, interactants joined each other (*N* = 238) on average 5.16 min (± 8.54) later.

### 3.3 Model 1a: Pant‐Hoot Type (IPH and Non‐IPH) Rates and Role of Social Factors

**Figure 3:**
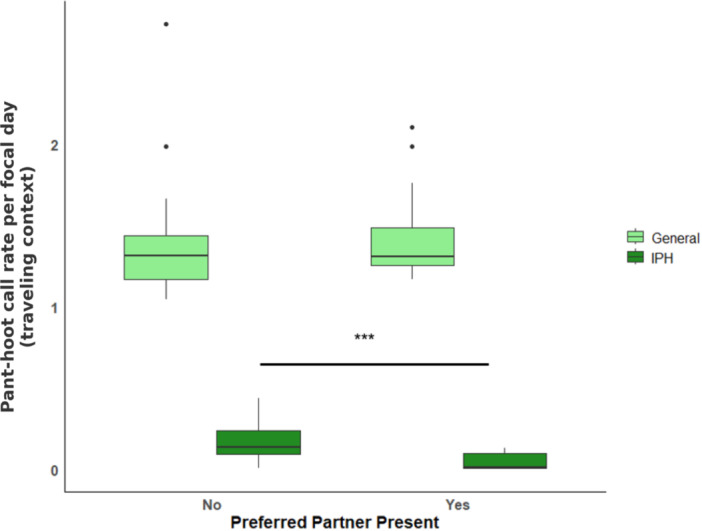
The figure shows the effect of preferred association partner presence on IPHs and general pant‐hoot call rate in the traveling context (GLMM; ***p ≤ 0.001). Boxes represent the median values for males with the upper and lower quartiles; whiskers represent data within 1.5 times the interquartile range.

We found that the full model differed significantly from the null model (LRT: *χ*² = 35.3, *df* = 5, *p* < 0.001). Social rank predicted pant‐hoot call rates, with higher‐ranking males producing more pant‐hoots (*β* = 0.56, SE = 0.17, 95% CI [0.23, 0.89], *χ*²(1) = 10.83, *p* = 0.001, Supporting Information S1: Table S2). In contrast, there was no evidence for an interaction between social rank and call type (*β* = −0.08, SE = 0.14, 95% CI [–0.35, 0.19], *χ*²(1) = 0.33, *p* = 0.57). We identified three key interactions involving pant‐hoot type. IPHs were produced more frequently in smaller parties (*β* = −0.45, SE = 0.14, 95% CI [–0.72, –0.18], *χ*²(1) = 9.00, *p* = 0.002) and during periods of high fission–fusion (*β* = 0.42, SE = 0.13, 95% CI [0.17, 0.67], *χ*²(1) = 6.64, *p* = 0.010). Finally, the presence of a preferred association partner differentially affected pant‐hoot production: IPHs were more common when the preferred partner was absent (*β* = −0.58, SE = 0.19, 95% CI [–0.95, –0.21], *χ*²(1) = 8.50, *p* = 0.003), whereas the presence of the partner did not affect the production of general pant‐hoots (*β* = 0.16, SE = 0.21, 95% CI [–0.25, 0.57], *χ*²(1) = 0.60, *p* = 0.440) (Figure 3).

### 3.4 Model 1b: Likelihood of Response

The full model differed significantly from the null model (LRT: *χ*² = 15.6, *df *= 3, *p* = 0.001). Social rank had a strong positive effect on the probability of receiving a response (*β* = 1.10, SE = 0.35, *χ*² = 9.90, *p* = 0.002), and social bond strength (dyadic) showed a modest effect (*β* = 0.85, SE = 0.40, *χ*² = 4.51, *df *= 1, *p* = 0.034), while caller age did not predict the likelihood of receiving a response (*β* = 0.30, SE = 0.28, *χ*² = 1.15, *df *= 1, *p* = 0.28, Supporting Information S1: Table S3).

### 3.5 Model 2: Intercall Times

**Figure 4:**
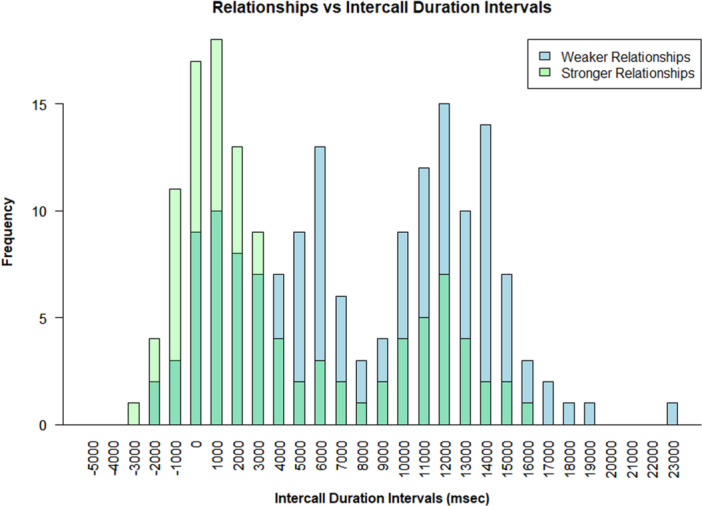
The figure depicts the response times to IPHs in ms as a function of relationship strength. Dyads considered to be strongly associated (i.e., above the 75% quartile) are displayed in green, and dyads considered to be weaker associated (i.e., below the 75% quartile) are depicted in blue.

The full model differed significantly from the null model (LRT: *χ*² = 22.4, *df *= 5, *p* = 0.0004). Higher DSI scores were associated with faster responses (*β* = −0.37, SE = 0.12, *p* = 0.004). By contrast, drumming presence significantly increased intercall intervals, resulting in longer response times (β = 0.52, SE = 0.15, *p* < 0.001, Supporting Information S1: Table S4). Neither the age of the caller (*β* = 0.08, SE = 0.11, *p* = 0.47) nor the age of the responder (*β* = 0.05, SE = 0.09, *p* = 0.61) was significantly associated with response times. There was no evidence for an interaction between social bond strength and the presence of drumming (*β* = 0.14, SE = 0.18, *p* = 0.42; Figure 4).

**Figure 5:**
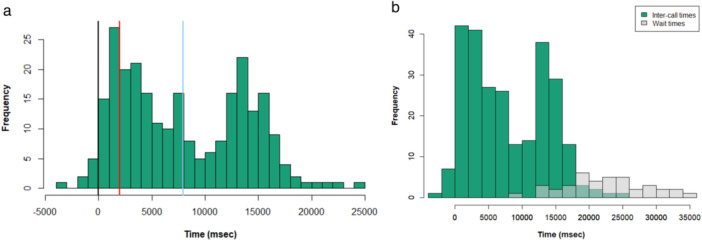
The figure shows (a) the frequency of intercall times in ms between an IPH and a response call. Intercall times are plotted along the *x*‐axis with three key markers in black, red, and blue. The black vertical line is at 0 ms, the red at 2000 ms, and the blue line marks the mean intercall duration of 7959 ms (± 5911). The black dotted line demarcates the boundary for calls with any overlap with the initiating pant‐hoot, the red line demarcates what is commonly accepted as the boundary for response times in previous studies concerning gestural transitions in chimpanzees (Fröhlich et al. 2016) and short‐range vocalizations in bonobos (Cornec et al. 2022). And (b) The frequency of intercall time durations (green) plotted against waiting time durations between two calls (marked in gray).

We plotted the frequency of response times for IPHs (*N* = 260) in 1 s (1000 ms) intervals across all observed dyads (Figure 5a). The response times ranged from −2.02 s to 25.12 s, and clustered around two distinct windows, an earlier interval of 0–5 s and alater interval of 12–16 s. Only 1.9% of IPH responses overlapped with the signalers initiating call. Seventy‐four % of the intercall times (197 of 260 cases) that exceeded the median duration of 7106 ms included response calls that were accompanied by a drumming bout. Furthermore, we identified a total of 32 IPH cases involving seven different dyads where an individual did not receive a response to his call and then called again. We refer to these events from here on as “multi‐call events.” Overall, the average waiting time between two subsequent IPH calls from the same emitter across the seven dyads was 22.36 ms ±7.2, ranging from 8.94 to 34.48 ms. The distribution of intercall intervals (*N* = 267) and waiting times (*N* = 32) differed significantly (Kolmogorov–Smirnov *D* = 0.78, *p* < 0.001), with waiting times being longer than intercall intervals (Figure 5b).

## 4 Discussion

This study aimed to provide a more comprehensive understanding of vocal turn‐taking in wild great apes by studying pant‐hoot production and exchanges in central chimpanzees in the wild. Overall, we found that the studied adult males produced approximately one pant‐hoot per hour, mostly while traveling or feeding. High‐ranking males called more frequently than lower‐ranking ones, especially when being in larger parties and during periods with high fission–fusion rates. About 44% of the calls produced in the travel context met the tested IPH criteria and were predominantly accompanied by waiting, scanning, drumming, and/or joining other group members shortly after call emission ( ~ 5 min). Around 75% received a vocal response, and approximately 20% of these included drumming. Concerning temporal patterns, we found that call response times of the chimpanzees to pant‐hoots were characterized by two distinct clusters, the first being between 0 and 5 s and the second between 12 and 16 s. In addition, dyads with stronger social bonds showed shorter response times, while the presence of drumming extended intercall intervals.

### 4.1 General Pant‐Hoot Patterns and Dynamics

Although few studies have systematically examined pant‐hoot context and call rates across chimpanzee sites and subspecies, our results align with several other studies (Clark and Wrangham 1993; Crunchant et al. 2021; Fedurek et al. 2016; Kalan 2019; Mitani and Nishida 1993). For instance, Western chimpanzee males of the South community, Taï National Park, Côte d'Ivoire, produced approximately 1.3 pant‐hoots per hour—closely matching our observed rate. In terms of context, pant‐hoots were most frequently produced during travel or feeding, a pattern also observed among Eastern chimpanzees in Mahale, Tanzania, Kanyawara, Kibale National Park and Sonso, Budongo Forest Reserve, Uganda (Fedurek et al. 2014; Mitani and Nishida 1993; Notman and Rendall 2005).

Caller identity also played a role in call frequency. As reported in several studies, high‐ranking males, especially alpha‐males, called more often (Clark and Wrangham 1994; Fedurek et al. 2014; Mitani and Nishida 1993), potentially reflecting several proposed functions of pant‐hooting, including heightened arousal, increased social motivation, and the vocal assertion of dominance or leadership. This status‐linked pattern extends beyond pant‐hoots: In both Taï and Kanyawara, high‐ranking males also produced food calls more frequently at feeding sites (Fedurek and Slocombe 2013; Kalan and Boesch 2015). Similar social status effects on calling patterns have been noted in other great apes (Mitani 1985; Luef et al. 2016) and also in some monkey species (e.g., *Papio cynocephalus ursinus*, Kitchen et al. 2003).

We also found that calling frequency increased with party size, echoing patterns from earlier studies (Crunchant et al. 2021; Goodall 1986; Reynolds and Reynolds 1965). However, this association is not consistent across all chimpanzee populations. For example, research at Kanyawara and Mahale did not find a significant link between party composition and pant‐hoot rate (Fedurek et al. 2014; Mitani and Nishida 1993), possibly indicating the influence of local ecology and culture. Finally, our findings on fission–fusion rates mirror those from a study at Kanyawara, where greater social fluidity (more frequent subgroup changes) was linked to increased vocal output (Fedurek et al. 2014), pointing to the important social‐cohesion function of these long‐distance calls.

### 4.2 Inquiring Pants‐Hoots

The males of the Rekambo community produced a distinct type of pant‐hoot during travel contexts, accompanied by specific behaviors such as actively listening and waiting for responses. Our findings offer empirical behavioral support for Goodall's long‐standing hypothesis that chimpanzees use IPHs to seek out and locate nearby group members. These results also add to the expanding body of work on interactional and turn‐taking capacities in nonhuman primates, highlighting its relevance not just for close‐range exchanges but also long‐distance communication (*Cebuella pygmaea*, Snowdon and Cleveland 1984; *Callithrix jacchus*, Takahashi et al. 2013; *Pan paniscus*, Schamberg et al. 2016). Moreover, they provide crucial evidence that chimpanzees engage in both gestural and structured vocal turn‐taking, challenging the idea that great apes are primarily specialized in gestural communication (Fröhlich et al. 2016; Badihi et al. 2024; Van Boekholt and Pika 2025; Levinson 2016).The present findings thus refute the idea that great apes primarily specialize in gestural turn‐taking. However, while our study did not compare vocal and gestural modalities directly, future research could investigate potential differences in flexibility, timing, and complexity between these communicative modes in both human and nonhuman great apes (Schamberg et al. 2016; Levréro et al. 2019).

### 4.3 The Role of Social Factors in IPHs

The Rekambo males emitted and received quicker responses to IPHs when traveling in smaller parties, during periods of increased fission–fusion events, and in the absence of preferred social partners. These findings deepen our understanding of the flexibility, goal‐directedness, and intentionality in chimpanzee long‐distance vocal behavior (Mitani and Brandt 1994; Girard‐Buttoz et al. 2022). Importantly, they complement observations of individuals from other communities where males increased call rates in the absence of key allies, presumably to re‐establish contact and coordinate reunions (Mitani and Nishida 1993; Bouchard and Zuberbühler 2022; Soldati et al. 2022). While accurately tracking all individuals' locations remains challenging, the context‐dependent nature of IPHs supports the view that such calls serve more than a simple broadcast function.

This targeted usage reinforces the idea that certain vocalizations, including pant‐hoots, act as functional analogs to grooming, serving to maintain cohesion and reinforce bonds in species with highly fluid social structures (Dunbar 1993; Fedurek et al. 2013; McComb and Semple 2005; Ramos‐Fernández 2005). Although pant‐hoots are more energetically costly than short‐range calls like grunts (baboons, Cheney et al. 1995), they are better suited to re‐establish bonds or coordinate movement across distance when visual contact is not possible. Our results may also imply that certain individuals were implicitly expected to respond. This idea, still speculative in the chimpanzee literature, echoes findings in dolphins, where signature whistles can function as individualized vocal addresses (Janik et al. 2006). The strong effect of social bond strength on intercall intervals may suggest that calls directed at close partners are not only more likely to receive a reply, but to do so promptly. Whether chimpanzees vocally tailor their calls or rely on shared history and social context to predict responses remains an open question.

Notably, IPH exchanges among the studied males showed minimal vocal overlap, echoing patterns characterizing human conversational turn‐taking (Sacks et al. 1974). They were also consistent with observations from the Kanyawara community(Arcadi 2000). These responses clustered within two distinct time windows: 0–5 s and 12–16 s. While humans typically respond within 1–2 s (Stivers et al. 2009), chimpanzee vocal exchanges appear to operate on a slower timescale. This pattern also diverges from great ape gestural exchanges, which often exhibit shorter response latencies (Fröhlich et al. 2016; Badihi et al. 2024). Still, the presence of clear, nonoverlapping windows suggests that turn‐taking in chimpanzees, though variable in duration, follows structured temporal norms across both vocal and gestural modalities. The second, delayed response window may still be interpreted by the caller as contingent, parsticularly since over seventy percent of responses after the median delay were accompanied by drumming. This behavior could reflect the time needed to identify a suitable tree for drumming, an action that may involve planning or more complex decision‐making processes (Goodall 1986; Fitzgerald et al. 2022). When further exploring chimpanzees' sensitivity to timing, we found that callers typically waited up to 20 s for a reply. This extended timeframe is in stark contrast to those commonly used in primate communication research to define “responses,” such as the 5 s window used by Arcadi (2000) tostudying vocalizations of Eastern chimpanzees or the 10 s criterion used by Schamberg and colleagues (2016) for bonobos. Our findings suggest that such cutoffs may strongly underestimate relevant responses. We propose that identifying response windows based on empirical distributions in the respective model species, rather than arbitrary thresholds, will provide a more species‐appropriate framework for studying vocal coordination and interactions in primates.

### 4.4 Limitations

Our research built upon Goodall's pioneering and foundational work (1986) to select and investigate IPHs; while this approach was very helpful in narrowing our scope, it resulted in excluding calls produced outside the travel context that may also be used by chimpanzees to inquire about other individuals. Moreover, the complexities of collecting data in the wild on naturally occuring behavior of wild chimpanzees traveling in separate groups presented significant challenges. For example, when researchers are in the field and can recognize the voices of all males, they begin to form a “mental map” of their locations. This mental map is not a precise tool for real‐time tracking but rather a conceptual aid to help understand the complexity of the fission–fusion society. For analysis purposes, however, the male potential audience was based on individuals not immediately present in the vocalizing individual's group. Future research would greatly benefit from improved communication systems among field observers, allowing for more comprehensive audio recordings capturing both sides of the interaction. Additionally, although our results suggest that IPHs are just a subset of pant‐hoots produced during travel and may serve a distinct function compared to pant‐hoots produced in other contexts, acoustic analyses are necessary to assess underlying acoustic structural differences. On this level, the integration of state‐of‐the‐art machine learning techniques could offer deeper insights into auditory variation within pant‐hoots across contexts. For example, recent studies have found further support for using structural acoustic features for context classification, with the number of let‐down elements being higher in traveling contexts (Desai et al. 2022; Fedurek et al. 2016). Finally, a larger data set containing more multicall events and dynamic “conversations” in the form of exchanged pant‐hoots would be ideal for further investigating information transfer and intricate aspects of turn‐taking frameworks, including the assignment of turns.

## 5 Conclusion

Overall, our study provided evidence that IPHs are a distinct form of pant‐hoots used to initiate “question–answer‐like” exchanges among Central African chimpanzees. They are used to maintain contact among group members, to initiate fusion events, and to initiate and nurture bonds among males, thus underscoring their role in shaping chimpanzees' spatial and social dynamics, but also possibly prosocial functions. While our findings are consistent with some findings of Arcadi's (2000) empirical observations—such as overlap patterns and the use of acoustically similar calls—they challenge his broader conclusion that pant‐hoot structures are of no special relevance to the evolution of linguistic systems. Instead, our results suggest that the observed exchanges of the Rekambo males followed structured, socially modulated temporal patterns, adding further fuel to the hypothesis that human cooperative turn‐taking may have evolved in the context of mutualistic prosocial activities.

## Author Contributions

**Lara Michelle Southern:** conceptualization (equal), data curation (lead), formal analysis (lead), investigation (lead), methodology (lead), visualization (equal), writing – original draft (lead), writing – review and editing (equal). **Tobias Deschner:** resources (equal), supervision (supporting), writing – review and editing (supporting). **Simone Pika:** idea, conceptualization (equal), funding acquisition (lead), resources (equal), supervision (lead), visualization (equal), shaping of the manuscript (lead), writing – review and editing (equal).

## Conflicts of Interest

The authors declare no conflicts of interest.

## Supporting information


**S1.** Demographic and observational summary of adult male chimpanzees in the Rekambo community (Loango National Park, Gabon). **S2.** Generalized Linear Mixed Model (GLMM) results (Model 1a): Predictors of pant‐hoot call rates in the travelling context. **S3.** GLMM results (Model 1b): Social predictors of vocal responses to Inquiring Pant‐Hoots. *Sample size:* N = 352 Inquiring pant‐hoots. **S4.** GLMM results (Model 2): Predictors of inter‐call intervals following Inquiring Pant‐Hoots that received a vocal response.

## Data Availability

The data supporting the findings of this study are available from the corresponding author upon request and will also be stored on the University Osnabrück Repository, available at: https://osnadocs.ub.uni-osnabrueck.de/.

## References

[ajp70092-bib-0001] Aamodt, C. M. , M. Farias‐Virgens , and S. A. White . 2020. “Birdsong as a Window Into Language Origins and Evolutionary Neuroscience.” Philosophical Transactions of the Royal Society, B: Biological Sciences 375, no. 1789: 20190060. 10.1098/rstb.2019.0060. PMC6895547 31735151

[ajp70092-bib-0002] Abreu, F. , and S. Pika . 2022. “Turn‐Taking Skills in Mammals: A Systematic Review Into Development and Acquisition.” Frontiers in Ecology and Evolution 10: 987253. 10.3389/fevo.2022.987253.

[ajp70092-bib-0101] Altmann, J. 1974. “Observational Study of Behavior: Sampling Methods.” Behaviour 49: 227–267. 4597405 10.1163/156853974x00534

[ajp70092-bib-0003] Arbib, M. A. , K. Liebal , and S. Pika . 2008. “Primate Vocalization, Gesture, and the Evolution of Human Language.” Current Anthropology 49, no. 6: 1053–1076. 10.1086/593015. 19391445

[ajp70092-bib-0005] Arcadi, A. C. 2000. “Vocal Responsiveness in Male Wild Chimpanzees: Implications for the Evolution of Language.” Journal of Human Evolution 39, no. 2: 205–224. 10.1006/jhev.2000.0415. 10968929

[ajp70092-bib-0008] Babiszewska, M. , A. M. Schel , C. Wilke , and K. E. Slocombe . 2015. “Social, Contextual, and Individual Factors Affecting the Occurrence and Acoustic Structure of Drumming Bouts in Wild Chimpanzees (*Pan troglodytes*).” American Journal of Physical Anthropology 156, no. 1: 125–134. 10.1002/ajpa.22634. 25327570

[ajp70092-bib-0009] Badihi, G. , K. E. Graham , C. Grund , et al. 2024. “Chimpanzee Gestural Exchanges Share Temporal Structure With Human Language.” Current Biology 34, no. 14: R673–R674. 10.1016/j.cub.2024.06.009. 39043136

[ajp70092-bib-0010] Bates, D. , M. Mächler , B. Bolker , and S. Walker . 2015. “Fitting Linear Mixed‐Effects Models Using lme4.” Journal of Statistical Software 67, no. 1: 1–48. 10.18637/jss.v067.i01.

[ajp70092-bib-0012] Van Boekholt, B. , and S. Pika . 2025. “Infrastructure of Mother–Infant Interactions Across Development in Chimpanzees (*Pan troglodytes*) in the Wild.” Evolution and Human Behavior 46: 106671. 10.1016/j.evolhumbehav.2025.106671.

[ajp70092-bib-0013] Boesch, C. 1991. “Symbolic Communication in Wild Chimpanzees?” Human Evolution 6, no. 1: 81–89. 10.1007/BF02435610.

[ajp70092-bib-0014] Bouchard, A. , and K. Zuberbühler . 2022. “Male Chimpanzees Communicate to Mediate Competition and Cooperation During Feeding.” Animal Behaviour 186: 41–55. 10.1016/j.anbehav.2022.01.009.

[ajp70092-bib-0016] Brima, Y. , L. Southern , U. Krumnack , G. Heidemann , and S. Pika . 2023. Individual Recognition in Wild Chimpanzees and Beyond: Supervised Representation Learning.” SSRN. 10.2139/ssrn.4520570.

[ajp70092-bib-0017] Cäsar, C. , and K. Zuberbühler . 2012. “Referential Alarm Calling Behaviour in New World Primates.” Current Zoology 58, no. 5: 680–697. 10.1093/czoolo/58.5.680.

[ajp70092-bib-0019] Cheney, D. L. , R. M. Seyfarth , and J. B. Silk . 1995. “The Role of Grunts in Reconciling Opponents and Facilitating Interactions Among Adult Female Baboons.” Animal Behaviour 50, no. 1: 249–257. 10.1006/anbe.1995.0237.

[ajp70092-bib-0021] Clark, A. P. , and R. W. Wrangham . 1993. “Acoustic Analysis of Wild Chimpanzee Pant Hoots: Do Kibale Forest Chimpanzees Have an Acoustically Distinct Food Arrival Pant Hoot?” American Journal of Primatology 31, no. 2: 99–109. 10.1002/ajp.1350310203. 31937003

[ajp70092-bib-0022] Clark, A. P. , and R. W. Wrangham . 1994. “Chimpanzee Arrival Pant‐Hoots: Do They Signify Food or Status?” International Journal of Primatology 15, no. 2: 185–205. 10.1007/BF02735273.

[ajp70092-bib-0023] Cornec, C. , M. Ngofuna , A. Lemasson , C. Monghiemo , V. Narat , and F. Levrero . 2022. “A Pilot Study of Calling Patterns and Vocal Turn‐Taking in Wild Bonobos *Pan paniscus* .” Ethology Ecology & Evolution 34, no. 3: 360–377. 10.1080/03949370.2022.2044387.

[ajp70092-bib-0025] Crockford, C. , I. Herbinger , L. Vigilant , and C. Boesch . 2004. “Wild Chimpanzees Produce Group‐Specific Calls: A Case for Vocal Learning?” Ethology 110, no. 3: 221–243. 10.1111/j.1439-0310.2004.00968.x.

[ajp70092-bib-0026] Crunchant, A. S. , F. A. Stewart , and A. K. Piel . 2021. “Vocal Communication in Wild Chimpanzees: A Call Rate Study.” PeerJ 9: e12326. 10.7717/peerj.12326. 34721995 PMC8532989

[ajp70092-bib-0027] Desai, N. P. , P. Fedurek , K. E. Slocombe , and M. L. Wilson . 2022. “Chimpanzee Pant‐Hoots Encode Individual Information More Reliably Than Group Differences.” American Journal of Primatology 84, no. 11: e23430. 10.1002/ajp.23430. 36093564 PMC9786991

[ajp70092-bib-0028] Dobson, A. J. , and A. G. Barnett . 2018. An Introduction to Generalized Linear Models (4th ed.). Chapman and Hall/CRC. 10.1201/9780429186624.

[ajp70092-bib-0029] Dunbar, R. I. M. 1993. “Coevolution of Neocortical Size, Group Size and Language in Humans.” Behavioral and Brain Sciences 16, no. 4: 681–694. 10.1017/S0140525X00032325.

[ajp70092-bib-0030] Eckhardt, N. , L. Polansky , and C. Boesch . 2015. “Spatial Cohesion of Adult Male Chimpanzees (*Pan troglodytes verus*) in Taï National Park, Côte d'Ivoire.” American Journal of Primatology 77, no. 2: 125–134. 10.1002/ajp.22323. 25256306

[ajp70092-bib-0102] Enfield, N. J. and Levinson, S. C. 2006. Roots of Human Sociality: Culture, Cognition and Interaction.

[ajp70092-bib-0031] Estienne, V. , B. Robira , R. Mundry , T. Deschner , and C. Boesch . 2019. “Acquisition of a Complex Extractive Technique by the Immature Chimpanzees of Loango National Park, Gabon.” Animal Behaviour 147: 61–76. 10.1016/j.anbehav.2018.11.002.

[ajp70092-bib-0032] Fedurek, P. , E. Donnellan , and K. E. Slocombe . 2014. “Social and Ecological Correlates of Long‐Distance Pant Hoot Calls in Male Chimpanzees.” Behavioral Ecology and Sociobiology 68, no. 8: 1345–1355. 10.1007/s00265-014-1745-4.

[ajp70092-bib-0033] Fedurek, P. , Z. P. Machanda , A. M. Schel , and K. E. Slocombe . 2013. “Pant Hoot Chorusing and Social Bonds in Male Chimpanzees.” Animal Behaviour 86, no. 1: 189–196. 10.1016/j.anbehav.2013.05.010.

[ajp70092-bib-0034] Fedurek, P. , and K. E. Slocombe . 2013. “The Social Function of Food‐Associated Calls in Male Chimpanzees.” American Journal of Primatology 75, no. 7: 726–739. 10.1002/ajp.22122. 23307442

[ajp70092-bib-0035] Fedurek, P. , K. Zuberbühler , and C. D. Dahl . 2016. “Sequential Information in a Great Ape Utterance.” Scientific Reports 6: 38226. 10.1038/srep38226. 27910886 PMC5133612

[ajp70092-bib-0036] Fitch, W. T. 2010. The Evolution of Language. Cambridge University Press.

[ajp70092-bib-0037] Fitzgerald, M. , E. P. Willems , A. Gaspard Soumah , T. Matsuzawa , and K. Koops . 2022. “To Drum or Not to Drum: Selectivity in Tree Buttress Drumming by Chimpanzees (*Pan troglodytes verus*) in the Nimba Mountains, Guinea.” American Journal of Primatology 84, no. 7: e23382. 10.1002/ajp.23382. 35383993 PMC9540414

[ajp70092-bib-0038] Fox, J. , and S. Weisberg . 2011. An R Companion to Applied Regression (2nd ed.). Sage Publications.

[ajp70092-bib-0039] Freeberg, T. M. , R. I. M. Dunbar , and T. J. Ord . 2012. “Social Complexity as a Proximate and Ultimate Factor in Communicative Complexity.” Philosophical Transactions of the Royal Society, B: Biological Sciences 367, no. 1597: 1785–1801. 10.1098/rstb.2011.0213. PMC3367695 22641818

[ajp70092-bib-0040] Fröhlich, M. , P. Kuchenbuch , G. Müller , et al. 2016. “Unpeeling the Layers of Language: Bonobos and Chimpanzees Engage in Cooperative Turn‐Taking Sequences.” Scientific Reports 6: 25887. 10.1038/srep25887. 27211477 PMC4876478

[ajp70092-bib-0103] Girard‐Buttoz, C. , E. Zaccarella , T. Bortolato , et al. 2022. “Chimpanzees Produce Diverse Vocal Sequences With Ordered and Recombinatorial Properties.” Communications Biology 5: 410. 10.1038/s42003-022-03350-8. 35577891 PMC9110424

[ajp70092-bib-0042] Goodall, J. 1986. The Chimpanzees of Gombe: Patterns of Behavior. Harvard University Press.

[ajp70092-bib-0045] Janik, V. M. , L. S. Sayigh , and R. S. Wells . 2006. “Signature Whistle Shape Conveys Identity Information to Bottlenose Dolphins.” Proceedings of the National Academy of Sciences 103, no. 21: 8293–8297. 10.1073/pnas.0509918103. PMC1472465 16698937

[ajp70092-bib-0046] Kalan, A. K. , and C. Boesch . 2015. “Audience Effects in Chimpanzee Food Calls and Their Potential for Recruiting Others.” Behavioral Ecology and Sociobiology 69: 1701–1712. 10.1007/s00265-015-1982-1.

[ajp70092-bib-0047] Kalan, A. K. 2019. “Evidence for Sexual Dimorphism in Chimpanzee Vocalizations: A Comparison of Male and Female Call Production and Acoustic Parameters.” In The Chimpanzees of the Taï Forest: 40 Years of Research, edited by C. Boesch and K. Zuberbühler , 410–421. Cambridge University Press.

[ajp70092-bib-0049] Kitchen, D. M. , R. M. Seyfarth , J. Fischer , and D. L. Cheney . 2003. “Loud Calls as Indicators of Dominance in Male Baboons (*Papio cynocephalus ursinus*).” Behavioral Ecology and Sociobiology 53, no. 6: 374–384. 10.1007/s00265-003-0588-1.

[ajp70092-bib-0104] Kolff, K. , and S. Pika . 2025. “Turn‐Taking in Grooming Interactions of Chimpanzees (*Pan troglodytes schweinfurthii*) in the Wild: The Role of Demographic and Social Factors.” Animal Cognition 28: 26. 10.1007/s10071-025-01940-7. 40126662 PMC11933235

[ajp70092-bib-0105] Lemasson, A. , H. Pereira , and F. Levréro . 2018. “Social Basis of Vocal Interactions in Western Lowland Gorillas (*Gorilla g. gorilla*).” Journal of Comparative Psychology 132, no. 2: 141–151. 10.1037/com0000105. 29528666

[ajp70092-bib-0050] Levinson, S. C. 2016. “Turn‐Taking in Human Communication: Origins and Implications for Language Processing.” Trends in Cognitive Sciences 20, no. 1: 6–14. 10.1016/j.tics.2015.10.010. 26651245

[ajp70092-bib-0051] Levinson, S. C. , and J. Holler . 2014. “The Origin of Human Multi‐Modal Communication.” Philosophical Transactions of the Royal Society, B: Biological Sciences 369, no. 1651: 20130302. 10.1098/rstb.2013.0302. PMC4123681 25092670

[ajp70092-bib-0052] Levréro, F. , S. Touitou , J. Frédet , B. Nairaud , J.‐P. Guéry , and A. Lemasson . 2019. “Social Bonding Drives Vocal Exchanges in Bonobos.” Scientific Reports 9: 711. 10.1038/s41598-018-36024-9. 30679444 PMC6346008

[ajp70092-bib-0053] Liebal, K. , B. M. Waller , A. M. Burrows , and K. E. Slocombe . 2013. Primate Communication: A Multimodal Approach. Cambridge University Press. 10.1177/147470491301100305 PMC10480985 23864293

[ajp70092-bib-0055] Luef, E. M. , T. Breuer , and S. Pika . 2016. “Food‐Associated Calling in Gorillas (*Gorilla g. gorilla*) in the Wild.” PLoS One 11, no. 2: e0144197. 10.1371/journal.pone.0144197. 26909518 PMC4766192

[ajp70092-bib-0056] McComb, K. , and S. Semple . 2005. “Coevolution of Vocal Communication and Sociality in Primates.” Biology Letters 1, no. 4: 381–385. 10.1098/rsbl.2005.0366. 17148212 PMC1626386

[ajp70092-bib-0058] Mitani, J. C. 1985. “Sexual Selection and Adult Male Orangutan Long Calls.” Animal Behaviour 33, no. 1: 272–283. 10.1016/S0003-3472(85)80063-4.

[ajp70092-bib-0059] Mitani, J. C. 2009. “Male Chimpanzees Form Enduring and Equitable Social Bonds.” Animal Behaviour 77: 633–640. 10.1016/j.anbehav.2008.11.021.

[ajp70092-bib-0060] Mitani, J. C. , and K. L. Brandt . 1994. “Social Factors Influence the Acoustic Variability in the Long‐Distance Calls of Male Chimpanzees.” Ethology 96, no. 3: 233–252. 10.1111/j.1439-0310.1994.tb01012.x.

[ajp70092-bib-0061] Mitani, J. C. , T. Hasegawa , J. Gros‐Louis , P. Marler , and R. Byrne . 1992. “Dialects in Wild Chimpanzees?” American Journal of Primatology 27, no. 4: 233–243. 10.1002/ajp.1350270402. 31941230

[ajp70092-bib-0062] Mitani, J. C. , K. L. Hunley , and M. E. Murdoch . 1999. “Geographic Variation in the Calls of Wild Chimpanzees: A Reassessment.” American Journal of Primatology 47, no. 2: 133–151. 10.1002/(SICI)1098-2345(1999)47:2<133::AID-AJP4>3.0.CO;2-I. 9973267

[ajp70092-bib-0063] Mitani, J. C. , and T. Nishida . 1993. “Contexts and Social Correlates of Long‐Distance Calling by Male Chimpanzees.” Animal Behaviour 45, no. 4: 735–746. 10.1006/anbe.1993.1088.

[ajp70092-bib-0064] Mitani, J. C. , and P. S. Rodman . 1979. “Territoriality: The Relation of Ranging Pattern and Home Range Size to Defendability, With an Analysis of Territoriality Among Primate Species.” Behavioral Ecology and Sociobiology 5, no. 3: 241–251. 10.1007/BF00293673.

[ajp70092-bib-0065] Mitani, J. C. , and J. Stuht . 1998. “The Evolution of Nonhuman Primate Loud Calls: Acoustic Adaptation for Long‐Distance Transmission.” Primates 39, no. 2: 171–182. 10.1007/BF02573076.

[ajp70092-bib-0067] Notman, H. , and D. Rendall . 2005. “Contextual Variation in Chimpanzee Pant Hoots and Its Implications for Referential Communication.” Animal Behaviour 70, no. 1: 177–190. 10.1016/j.anbehav.2004.08.024.

[ajp70092-bib-0069] Partan, S. , and P. Marler . 1999. “Communication Goes Multimodal.” Science 283, no. 5406: 1272–1273. 10.1126/science.283.5406.1272. 10084931

[ajp70092-bib-0071] Pika, S. , R. Wilkinson , K. H. Kendrick , and S. C. Vernes . 2018. “Taking Turns: Bridging the Gap Between Human and Animal Communication.” Proceedings of the Royal Society B: Biological Sciences 285, no. 1880: 20180598. 10.1098/rspb.2018.0598. PMC6015850 29875303

[ajp70092-bib-0074] Pougnault, L. , F. Levréro , B. Mulot , and A. Lemasson . 2020. “Breaking Conversational Rules Matters to Captive Gorillas: A Playback Experiment.” Scientific Reports 10: 6947. 10.1038/s41598-020-63923-7. 32332855 PMC7181860

[ajp70092-bib-0106] R Core Team . 2022. R: A Language and Environment for Statistical Computing. R Foundation for Statistical Computing.

[ajp70092-bib-0075] Ramos‐Fernández, G. 2005. “Vocal Communication in a Fission–Fusion Society: Do Spider Monkeys Stay in Touch With Close Associates?” International Journal of Primatology 26: 1077–1092. 10.1007/s10764-005-6459-z.

[ajp70092-bib-0077] Reynolds, V. , and F. Reynolds . 1965. “Chimpanzees of the Budongo Forest.” In Primate Behavior: Field Studies of Monkeys and Apes, edited by I. DeVore , 368–424. Holt, Rinehart and Winston.

[ajp70092-bib-0078] Rosati, A. G. , Z. P. Machanda , and K. E. Slocombe . 2022. “Cognition in the Wild: Understanding Animal Thought in Its Natural Context.” Current Opinion in Behavioral Sciences 47: 101210. 10.1016/j.cobeha.2022.101210.

[ajp70092-bib-0079] Rossano, F. 2013. “Asymmetry and Adaptation in Social Interaction: A Micro‐Analytic Perspective.” Interaction Studies. Social Behaviour and Communication in Biological and Artificial Systems 14, no. 2: 160–189. 10.1075/is.14.2.02ros.

[ajp70092-bib-0080] Rossano, F. , and K. Liebal . 2014. “‘Requests’ and ‘Offers’ in Orangutans and Human Infants.” In Requesting in Social Interaction, edited by P. Drew and E. Couper‐Kuhlen , 335–364. John Benjamins Publishing Co. 10.1075/slsi.26.13ros.

[ajp70092-bib-0081] Sacks, H. , E. A. Schegloff , and G. Jefferson . 1974. “A Simplest Systematics for the Organization of Turn‐Taking for Conversation.” Language 50, no. 4: 696–735. 10.2307/412243.

[ajp70092-bib-0082] Schamberg, I. , D. L. Cheney , Z. Clay , G. Hohmann , and R. M. Seyfarth . 2016. “Call Combinations, Vocal Exchanges and Interparty Movement in Wild Bonobos.” Animal Behaviour 122: 109–116. 10.1016/j.anbehav.2016.10.003.

[ajp70092-bib-0083] Searcy, W. A. , and M. Andersson . 1986. “Sexual Selection and the Evolution of Song.” Annual Review of Ecology and Systematics 17: 507–533. 10.1146/annurev.es.17.110186.002451.

[ajp70092-bib-0084] Seyfarth, R. M. , and D. L. Cheney . 2003. “Signalers and Receivers in Animal Communication.” Annual Review of Psychology 54: 145–173. 10.1146/annurev.psych.54.101601.145121. 12359915

[ajp70092-bib-0085] Silk, J. B. , S. C. Alberts , and J. Altmann . 2003. “Social Bonds of Female Baboons Enhance Infant Survival.” Science 302: 1231–1234. 10.1126/science.1088580. 14615543

[ajp70092-bib-0086] Snowdon, C. T. , and J. Cleveland . 1984. “‘Conversations’ Among Pygmy Marmosets.” American Journal of Primatology 7, no. 1: 15–20. 10.1002/ajp.1350070104. 32138463

[ajp70092-bib-0087] Soldati, A. , P. Fedurek , G. Dezecache , J. Call , and K. Zuberbühler . 2022. “Audience Sensitivity in Chimpanzee Display Pant Hoots.” Animal Behaviour 190: 23–40. 10.1016/j.anbehav.2022.05.010.

[ajp70092-bib-0088] Stivers, T. , N. J. Enfield , P. Brown , et al. 2009. “Universals and Cultural Variation in Turn‐Taking in Conversation.” Proceedings of the National Academy of Sciences 106, no. 26: 10587–10592. 10.1073/pnas.0903616106. PMC2705608 19553212

[ajp70092-bib-0089] Takahashi, D. Y. , D. Z. Narayanan , and A. A. Ghazanfar . 2013. “Coupled Oscillator Dynamics of Vocal Turn‐Taking in Monkeys.” Current Biology 23: 2162–2168. 10.1016/j.cub.2013.09.005. 24139740

[ajp70092-bib-0090] Teixeira da Cunha, R. G. , and R. W. Byrne . 2009. “The Use of Vocal Communication in Keeping the Spatial Cohesion of Groups: Intentionality and Specific Functions.” In South American Primates: Comparative Perspectives in the Study of Behavior, Ecology, and Conservation, edited by P. A. Garber , A. Estrada , J. C. Bicca‐Marques , E. W. Heymann and K. B. Strier , 341–363. Springer. 10.1007/978-0-387-78705-3_18.

[ajp70092-bib-0091] Uhlenbroek, C. 1995. “The Structure and Function of the Long‐Distance Calls Given by Male Chimpanzees in Gombe National Park.” Doctoral Dissertation, University of Bristol.

[ajp70092-bib-0092] de Vries, H. , W. J. Netto , and P. L. H. Hanegraaf . 1993. “MatMan: A Program for the Analysis of Sociometric Matrices and Behavioural Transition Matrices.” Behaviour 125, no. 3–4: 157–175. 10.1163/156853993X00218.

[ajp70092-bib-0093] de Vries, H. , J. M. G. Stevens , and H. Vervaecke . 2006. “Measuring and Testing the Steepness of Dominance Hierarchies.” Animal Behaviour 71, no. 3: 585–592. 10.1016/j.anbehav.2005.05.015.

[ajp70092-bib-0095] White, F. J. , M. Waller , K. Boose , M. Y. Merrill , and K. D. Wood . 2015. “Function of Loud Calls in Wild Bonobos.” Journal of Anthropological Sciences 93: 1–13. 10.4436/JASS.93003. 25324464

[ajp70092-bib-0096] Wich, S. , and C. Nunn . 2002. “Do Male ‘Long‐Distance Calls’ Function in Mate Defense? A Comparative Study of Long‐Distance Calls in Primates.” Behavioral Ecology and Sociobiology 52, no. 6: 474–484. 10.1007/s00265-002-0541-8.

[ajp70092-bib-0097] Seyfarth, R. M. , D. L. Cheney , and T. J. Bergman . 2005. “Primate Social Cognition and the Origins of Language.” Trends in Cognitive Sciences 9, no. 6: 264–266. 15925802 10.1016/j.tics.2005.04.001

