# Supplementary material for: Inquiring Pant‐Hoots in Wild Chimpanzees and the Role of Social Bonds and Group Cohesion
Source: Am J Primatol. 2025 Dec 12;87(12):e70092. doi: 10.1002/ajp.70092 (PMC12699367; doi:10.1002/ajp.70092)
Supplement: Supplementary file 1 — S1. Demographic and observational summary of adult male chimpanzees in the Rekambo community (Loango National Park, Gabon). S2. Generalized Linear Mixed Model (GLMM) results (Model 1a): Predictors of pant‐hoot call rates in the travelling context. S3. GLMM results (Model 1b): Social predictors of vocal responses to Inquiring Pant‐Hoots. Sample size: N = 352 Inquiring pant‐hoots. S4. GLMM results (Model 2): Predictors of inter‐call intervals following Inquiring Pant‐Hoots that received a vocal response. [file AJP-87-e70092-s001.docx]

***Supporting Information***

Inquiring Pant-Hoots' in Chimpanzees in the Wild: Social Bonds and Group Cohesion

Lara Michelle Southern^1^*, Tobias Deschner^1,2^, Simone Pika^1^

^1^Comparative BioCognition, Institute of Cognitive Science, Osnabrück University, Osnabrück,

Germany

^2^Max Planck Institute for Evolutionary Anthropology, Leipzig, Germany

**Corresponding author:**

Lara Michelle Southern

lara.southern@gmail.com

**This file includes:**

Supplementary methods section

Tables S1-S4

**Methods**

**Identity recognition.** To ensure reliable individual identification of pant-hoots, LMS underwent a structured training period during a three-month pilot study. During this phase, she systematically recorded pant-hoots from all adult males across a range of acoustic conditions, including different habitats (e.g., closed-canopy forest, coastal forest, and savannah) and varying background noise levels (e.g., insect choruses, wind, distant calls). Recordings were made using a Sennheiser ME66/K6 directional microphone connected to a Zoom H4n recorder (44.1 kHz, 16-bit). While conducting focal sampling, we aimed to record pant-hoots within approximately 30 meters of the focal individual to ensure clarity and to create a high-quality reference library for each individual. However, during the training phase, LMS also listened to and practiced classification on pant-hoots recorded at longer distances—up to approximately 500 meters—to increase familiarity with individual variation under more degraded acoustic conditions. In the field during this period, the identity of the caller was also visually verified either by LMS or a second team using GPS radio coordination. To assess reliability at the end of the training period, we conducted an intra-rater reliability test. A subset of pant-hoots (at least 6 per individual) was relabeled in a blind test setup coordinated by the research manager. LMS independently reassigned caller identities to these recordings, and the predicted labels were compared to the confirmed identities. Agreement was quantified using Cohen’s Kappa and weighted Kappa, calculated with the “kappa2” function in the “irr” package in R (Gamer et al., 2012). The resulting score of 0.95 indicates an 'almost perfect' level of agreement (McHugh, 2012), confirming the high accuracy of individual recognition. That said, we acknowledge certain limitations. While LMS trained with a broader range of recordings—including those recorded at distances up to 500 meters— we did not control of the distance or acoustic quality specifically in the calls used for the intra-rater reliability test. Future studies incorporating more automated acoustic feature analysis or playback validation would help further strengthen assessments of vocal individual recognition in field conditions.

**Pant-hoot recording.** For calls recorded without audio equipment, pant-hoot exchanges were logged using Cybertracker, a stop-go method validated by an intra-rater reliability test yielding an agreement score of 0.79. Only calls that were mostly isolated (minimal overlap or part of chorusing bout), free of high levels of background noise, and where the caller’s identity and behavior were confirmed, were included in the analysis. Acoustic data extraction was performed using Praat software (Boersma, 2001). After each pant-hoot recording, responses were noted, and the distance between the caller and responder was estimated. When two research teams were present and one team was with the responder, both estimated and true distances were recorded using a GPS feature (Garmin Rino 700) (N=68) to test reliability. This yielded an intra-rater reliability score of 0.74, indicating moderate agreement (McHugh, 2012), supporting our use of estimated distances as true values.

**Speed of sound adjustment.** Given that sound travels at a finite speed, we always estimated the distance between the caller and the responder and standardized our calculations to 350 m/s, thereby consistently accounting for the delay in response time due to sound travel (Naguib & Wiley, 2001). This adjustment allowed us to better determine when individuals processed the calls they heard and how their response calls in turn traveled over distances.

**Dyadic Composite Sociality Index.**

DSI = $\left\{ \frac{\left( G_{A+B}/{(G}_{A+B}+G_{A}+G_{B})+(P_{A+B}/{(P}_{A+B}+P_{A}+P_{B} \right)}{2} \right\}$

Where G_A+B_ is the frequency of grooming role of individual _A_ towards individual _B_ and vice versa. G_A_ represents the total frequency of grooming roles observed of individual A in the absence of individual B, and G_B_ is the total frequency of individual B in the absence of individual A. Similarly, P_A+B_ is the number of scans where individuals A and B were observed to be in proximity to each other (10m). P_A_ and P_B_ stand for the total number of scans for individuals A or B, excluding the other individual (A/B). Each individual had a ‘preferred association partner’ indicated by the highest DSI value.

**Supplementary Tables**

**S1.** **Demographic and observational summary of adult male chimpanzees in the Rekambo community (Loango National Park, Gabon).**

*Adult males of the Rekambo community listed in alphabetical order with information pertaining to name, three-digit abbreviation (ID code), age (in years), rank standing as per the period 2018-2020, total observation hours and total number of pant hoot calls emitted.*

| **Name**  **(ID code)** | **Age (estimate in 2020)** | **Rank (1 = highest)** | **Total observation hours** | **Total pant-hoot calls** |
| --- | --- | --- | --- | --- |
| ARNOLD (ARN) | 17 | 10 | 102 | 191 |
| CHENGE (CHE) | 24 | 5 | 112 | 144 |
| CHINOIS (CHI) | 28 | 2 | 131 | 205 |
| FREDDY (FRE) | 22 | 4 | 199 | 198 |
| LITTLEGREY (LIT) | 20 | 7 | 129 | 165 |
| LOUIS (LOU) | 27 | 3 | 103 | 158 |
| NGONDE (NGO) | 19 | 8 | 79 | 189 |
| ORION (ORI) | 18 | 9 | 69 | 104 |
| PANDI (PAN) | 22 | 1 | 214 | 245 |
| THEA (THE) | 21 | 6 | 182 | 149 |

**Model Tables**

**S2. Generalized Linear Mixed Model (GLMM) results (Model 1a): Predictors of pant-hoot call rates in the travelling context.**

**Sample size:** *N = 301 focal observations.*

Call rate per focal day was modeled as a function of social and contextual predictors.
A total of 789 travel pant-hoots were recorded across 301 focal days.

| **Parameter** | **β (Estimate)** | **SE** | **Lower 95% CI** | **Upper 95% CI** | **χ²** | **p** | **Min** | **Max** |
| --- | --- | --- | --- | --- | --- | --- | --- | --- |
| Social Rank | 0.56 | 0.17 | 0.23 | 0.89 | 10.83 | 0.001 | 0.45 | 0.65 |
| Rank × Call Type | -0.08 | 0.14 | -0.35 | 0.19 | 0.33 | 0.57 | -0.20 | 0.10 |
| Party Size × Call Type | -0.45 | 0.14 | -0.72 | -0.18 | 9.00 | 0.002 | -0.60 | -0.30 |
| Fission-Fusion Rate × Call Type | 0.42 | 0.13 | 0.17 | 0.67 | 6.64 | 0.010 | 0.30 | 0.55 |
| Preferred Partner (IPH) | -0.58 | 0.19 | -0.95 | -0.21 | 8.50 | 0.003 | -0.70 | -0.45 |
| Preferred Partner (GPH) | 0.16 | 0.21 | -0.25 | 0.57 | 0.60 | 0.440 | 0.05 | 0.27 |

Note: Call Type refers to Inquiring Pant-Hoot (IPH) vs. General Pant-Hoot (GPH)

**S3. GLMM results: (Model 1b): Social predictors of vocal responses to Inquiring Pant-Hoots**

**Sample size:** *N = 352 Inquiring pant-hoots*

| **Parameter** | **β (Estimate)** | **SE** | **Lower 95% CI** | **Upper 95% CI** | **χ²** | **p** |
| --- | --- | --- | --- | --- | --- | --- |
| Social Rank | 1.10 | 0.35 | 0.41 | 1.79 | 9.90 | 0.002 |
| Social Bond Strength | 0.85 | 0.40 | 0.07 | 1.63 | 4.51 | 0.034 |
| Age (Caller) | 0.30 | 0.28 | -0.25 | 0.85 | 1.15 | 0.28 |

**S4. GLMM results: (Model 2): Predictors of inter-call intervals following Inquiring Pant-Hoots that received a vocal response**

**Sample size:** *N = 260 inter-call intervals (from calls that received a response)*

| **Parameter** | **β (Estimate)** | **SE** | **Lower 95% CI** | **Upper 95% CI** | **p** |
| --- | --- | --- | --- | --- | --- |
| Association Scores | -0.37 | 0.12 | -0.61 | -0.13 | 0.004 |
| Drumming Presence | 0.52 | 0.15 | 0.23 | 0.81 | <0.001 |
| Age (Caller) | 0.08 | 0.11 | -0.13 | 0.29 | 0.47 |
| Age (Responder) | 0.05 | 0.09 | -0.12 | 0.22 | 0.61 |
| Association × Drumming | 0.14 | 0.18 | -0.21 | 0.49 | 0.42 |

**References**

Boersma, P. (2001). Praat, a system for doing phonetics by computer. Glot. Int., 5(9), 341-345.

McHugh, M. L. (2012). Interrater reliability: the kappa statistic. *Biochemia medica*, 22(3), 276-282. https://pubmed.ncbi.nlm.nih.gov/23092060.

Naguib, M., & Wiley, R. H. (2001, 2001/11/01/). Estimating the distance to a source of sound: mechanisms and adaptations for long-range communication. *Animal behaviour*, 62(5), 825-837. 10.1006/anbe.2001.1860.
